# Determinants of sodium intake knowledge and attitude: a cross-national analysis of socio-economic and health factors

**DOI:** 10.1017/S1368980024001551

**Published:** 2024-10-22

**Authors:** Santosh Kumar Rauniyar, Yuta Tanoue, Cyrus Ghaznavi, Hitomi Hayabuchi, Toshihide Nishimura, Yukari Takemi, Shuhei Nomura

**Affiliations:** 1 Department of Global Health Policy, Graduate School of Medicine, The University of Tokyo, Tokyo, Japan; 2 Department of Health Policy and Management, School of Medicine, Keio University, Tokyo, Japan; 3 Ocean Research Policy Institute, The Sasakawa Peace Foundation, Tokyo, Japan; 4 Faculty of Marine Technology, Tokyo University of Marine Science and Technology, Tokyo, Japan; 5 Graduate School of Health and Environmental Sciences, Fukuoka Women’s University, Fukuoka, Japan; 6 Faculty of Nutrition, Kagawa Nutrition University, Saitama, Japan; 7 Keio University Global Research Institute (KGRI), Tokyo, Japan

**Keywords:** Knowledge about Na intake, Attitude towards Na intake, Health condition and Na consumption, Awareness level

## Abstract

**Objective:**

The aim of this study is to conduct a comparative analysis across nations to: (1) identify the determinants influencing knowledge and attitudes related to sodium (Na) intake and (2) to analyse the association between knowledge and attitudes related to Na intake.

**Design:**

We utilised a secondary data from a cross-sectional study that was conducted across seven nations. Structural equation modelling (SEM) was utilised to assess the impact of socio-economic and health-related predictors on knowledge and attitudes pertaining to Na intake and further to investigate the relationship between knowledge and attitude.

**Setting:**

Indonesia, Brazil, Thailand, Japan, France, the UK and the USA.

**Participants:**

7090 participants aged 15 years and above were included in the study.

**Results:**

SEM analysis showed a strong association between knowledge about Na intake and related attitude across all countries, particularly in the UK (2·65, 95 % CI 1·48–3·82), France (2·62, 1·45–3·79) and the USA (1·97, 1·21–2·73). In Brazil, Japan and France, individuals or family members having certain health conditions such as raised blood pressure, heart diseases, strokes or other diseases exhibited a positive attitude towards reducing Na intake. Conversely, socio-economic factors like education and income demonstrated the complexity of influences on knowledge and attitudes about Na intake.

**Conclusion:**

The study underscores the need for tailored public health interventions to reduce excessive Na consumption, considering the diverse cultural, social and economic factors. It highlights the complex determinants of knowledge and attitudes towards Na intake, calling for further research in varied populations.

Excessive Na intake is a significant public health concern around the globe as it has been linked to chronic non-communicable diseases (NCD), such as hypertension, heart disease and stroke^(1,2)^. Although a certain amount of Na consumption daily is required, excessive intake may cause health complications^(3)^. For instance, Na affects fluid regulation; thus, high Na intake may increase blood pressure through volume expansion^(4)^. It is estimated that about 1·28 billion adults aged 30–79 years worldwide have been diagnosed with hypertension^(5)^, and high Na intake is a major risk factor for the development of high blood pressure^(6)^. Taking into account the need to avoid excessive Na consumption that may precipitate CVD, the WHO has recommended approximately 2000 milligrams per d Na intake^(7)^. However, according to the WHO global report on Na intake reduction 2023, the global mean population Na intake in 2019 was estimated to be 4310 mg/d with wide variation in consumption across countries^(8)^.

In 2005, the World Action of Salt, Sugar, and Health (WASSH) was established to encourage a worldwide reduction in Na intake. The WHO’s action plan included five key components: surveillance, product reformulation, standardised food labelling, knowledge and environment^(9)^. In support of this action plan, in 2013, the World Health Assembly (WHA) committed to nine global voluntary targets to reduce NCD and a 30 % relative reduction in Na intake in the population by 2025^(7)^. However, as of 2021, not a single country among the 194 WHO member countries has been able to achieve the recommended Na intake reduction target^(10)^.

The interplay between knowledge and attitudes related to Na consumption is a pivotal determinant of dietary behaviours, significantly impacting public health by influencing the incidence of chronic NCD. While the association between limited Na-related knowledge, suboptimal attitudes towards Na consumption and the challenges in curbing excessive intake has been observed in various contexts, research specifically addressing these issues remains sporadic and is not uniformly distributed across the globe^(11–14)^. Notably, empirical studies, such as the cross-sectional survey in Los Angeles, the USA, intervention research in Lebanon and Na reduction programmes in China, have underscored the beneficial impact of enhancing Na-related knowledge and attitudes on intake reduction^(15–17)^. These findings highlight the essential role of informed public health interventions in lessening the NCD burden linked to high Na consumption. Nonetheless, a significant literature gap exists in comprehensive cross-national analyses, particularly in examining the socio-economic and health-related determinants of Na knowledge and attitudes^(18,19)^.

Addressing this deficiency, the present study aims to offer a comparative analysis to decipher the socio-economic and health-related predictors influencing knowledge and attitude related to Na intake and analyse the association between Na knowledge and attitudes across the aforementioned nations, utilising structural equation modeling (SEM). By investigating how knowledge and attitudes towards Na intake diverge across varied cultural and socio-economic backdrops, this research intends to furnish a detailed understanding of the multifaceted global challenge of Na intake reduction. The insights derived from this study are expected to not only fill a critical gap in the cross-national comparative literature but also steer the formulation of nuanced and effective public health strategies tailored to the unique contexts of these countries, thereby significantly contributing to the global effort to mitigate excessive Na consumption and diminish the prevalence of NCD worldwide.

## Method

We used data obtained from a survey commissioned by Ajinomoto Co., Inc., and conducted by Edelman Data & Intelligence (USA), a company that provides internet survey services and has a worldwide panel of respondents owned by Edelman Data and Intelligence. The online survey was conducted between 31 August and 24 September 2021, and included questions related to Na perception that specifically measured knowledge and attitude of participants related to Na intake along with their demographics and socio-economic characteristics. Participation in the survey was voluntary, and responses were anonymised. The questionnaire for the survey was drafted by Edelman Data and Intelligence based on their experience and expertise in conducting such types of surveys and further extensively reviewed by the experts of food and nutrition science at Ajinomoto Co. There were no specific inclusion or exclusion criteria stated; however, the study participants were aged 15 years and over. A quota sampling method was used based on gender, age and region to ensure the national representation of each country in the sample. The survey closed after reaching a predetermined sample size set at approximately 1000 respondents for each country considering the time and resources availability. The study included 7090 respondents from seven countries: Indonesia, Brazil, Thailand, Japan, France, the UK and the USA. These countries were selected based on data accessibility and taking into account various socio-economic backgrounds and different food cultures to ensure regional diversity.

### Study design

Our analysis primarily focused on two latent outcome variables: (i) knowledge regarding Na intake (referred to as ‘knowledge’ hereafter) and (ii) attitude towards reducing excessive Na intake (referred to as ‘attitude’ hereafter). Latent variables are theoretical constructs that are not directly measurable but can be estimated through mathematical models using observed variables. These observed variables, often referred to as items/indicators, are used to quantify the latent construct^(20)^. Confirmatory factor analysis (CFA) was utilised to identify the significant observed variables/items measuring the two latent outcome variables. A detailed account of the selection process for these observed variables/items through CFA is available in Appendix 1·1 and 1·2.

**Fig. 1:**
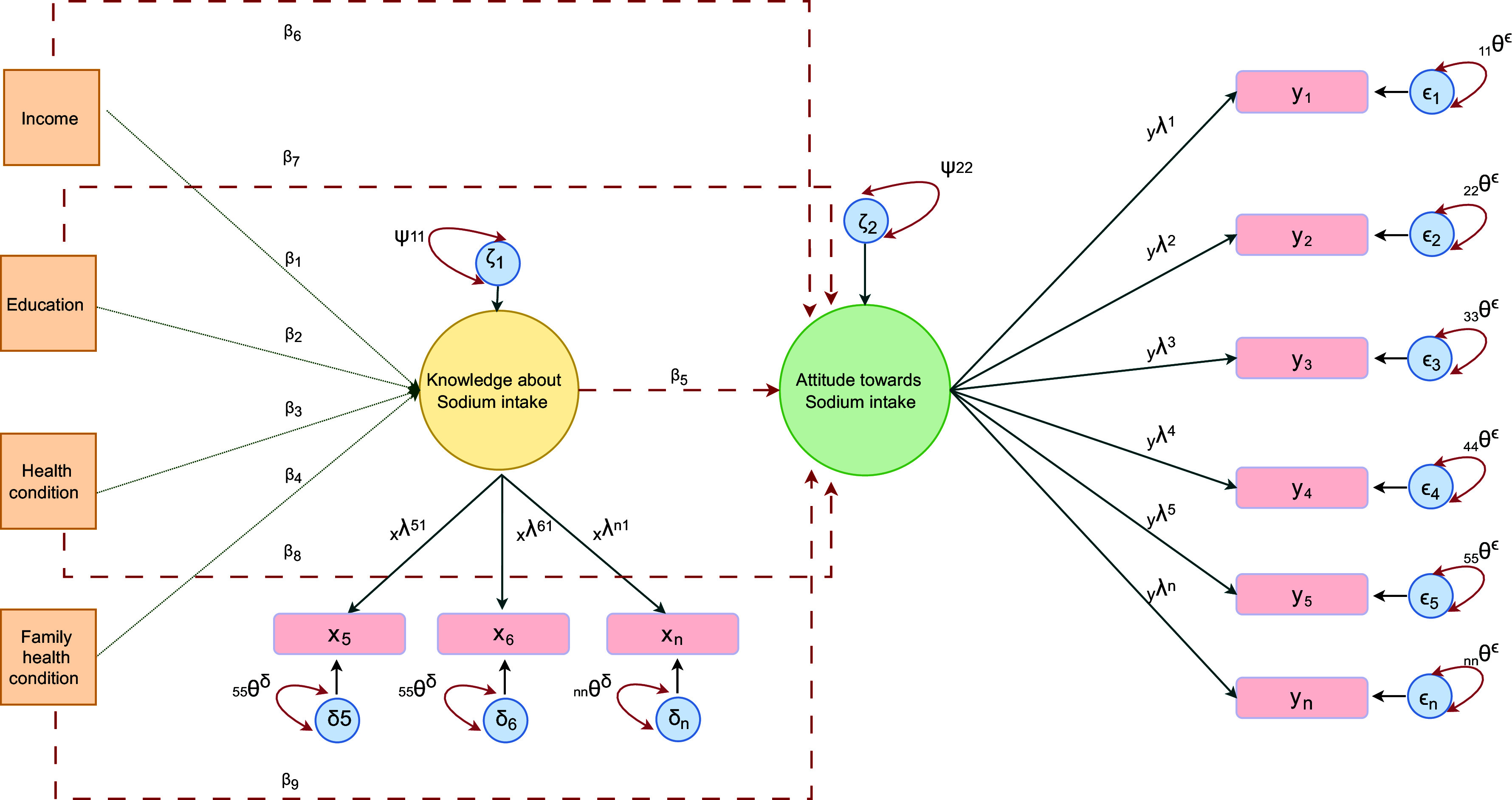
Conceptual frame of structural equation model.* *x* and *y* – observed variables; 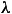 – loading scores; 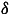 and 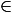 – residuals of observed variables; 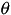 – variance; 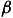 – regression coefficient; 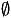 – residual of exogenous variables; 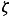 – residual of endogenous variable; 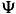 – residual variance

Following the identification of significant observed variables, that is, items/indicators, we employed SEM to analyse the influence of socio-economic and health determinants (denoted as exogenous variables in SEM), income, education, health condition and family health condition on the outcome variables (termed as endogenous variables in SEM), knowledge and attitude, for each country separately. Furthermore, in the regression equation of outcome variable ‘attitude’, knowledge was treated as a predictor variable (exogenous variable) to examine the association with attitude (Fig. 1). To facilitate comparison among countries, the income variable was adjusted to average gross domestic product (GDP) per capita of the respective country and reclassified into three categories: income level below the average GDP per capita, income level equal to average GDP per capita and income level above the average GDP per capita. The variables ‘individual health conditions’ and ‘family health conditions’ had several categories based on presence or absence of the disease types (e.g. hypertension, stroke, heart disease, kidney disease, stomach cancer, other and none of the above). Thus, these two variables were reclassified into two categories: been diagnosed by health professional and not been diagnosed by the health professional for the above-mentioned health issues.

### Structural equation model

SEM combines factor analysis principles with path analysis modelling methods in specifying a set of linear equations representing hypothesised relations among latent constructs and their multiple indicators^(21)^. The general SEM framework, as outlined by Jöreskog (1973), consist of two interrelated components: (i) a measurement model and (ii) a structural model^(22)^. The measurement model specifies how latent construct is measured by observed indicators and describes the measurement properties (reliability and validity) of the indicators, which is analogous to CFA. The structural model specifies causal relationship among the latent variables, describes their direct and indirect effects, and allocates explained and unexplained variances of the dependent constructs^(21,23)^ The conceptual framework of SEM is shown in Fig. 1, and the explanation of SEM is provided in Appendix 1·2.

The systems of simultaneous equations for SEM are as follows:

The latent variables are linked to observable variables via measurement equations for the endogenous variables and exogenous variables. These equations are defined as:

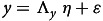




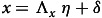

where *x* is the observed indicator of endogenous latent variables knowledge and attitude and *y* is the observed indictors of exogenous latent variable (predictors), income, education, health condition and family health condition. 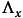 and 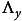 are matrices of factor loadings, respectively. 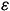 and 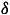 are vector of uniqueness, respectively. In addition, the general model specifies variances are covariances for 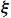 , 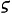 , 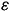 and 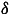 , denoted by 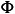 , 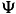 , 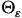 and 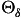 , respectively^(22)^ (Fig. 1).

The structural part of the model can be written as:

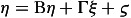

where 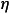 is a vector of endogenous (criterion) latent variables. 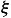 is a vector of exogenous (predictor) latent variables. 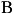 is a matrix of regression coefficients relating the latent endogenous variables to each other, 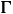 is a matrix of regression coefficients relating endogenous variables to exogenous variables and 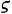 is a vector of disturbance terms. The detail equation of SEM model is provided in appendix. The final structural equation model for each country was selected based on the comparative fit index and Tucker–Lewis index scores provided in Supplementary Table 5. The details of the survey questions that comprised the measured variables, latent construct and the results of the CFA are provided in Supplementary Tables 1–3.

## Results

Table 1 provides the demographic information of 7090 participants from seven countries (Indonesia, Brazil, Thailand, Japan, France, the UK and the USA), including income, education, age, gender and health status. Two-thirds of the participants from Indonesia, Brazil, Thailand, Japan and the UK had income levels equivalent to or higher than the GDP per capita of their respective countries. Most of the participants included in the study had higher/university level education with the highest percentage of respondents having higher/university level education in the USA (72·5 %), followed by both Japan and Thailand (57·6 %), Brazil (56·9 %), Indonesia (56·5 %), France (46·3 %), and the UK (46·3 %). Furthermore, 60 % of the total study participants in two countries with lower GDP (Indonesia and Brazil) were below 35 years of age.

**Table 1:** Summary of characteristics of the participants

Country	Indonesia		Brazil		Thailand		Japan		France		The UK		The USA	
No of participants	1015		1026		1021		1000		1006		1022		1000	
GDP per capita (2019 USD)*	12358·2		15304·9		18763·5		42282·6		48971·1		49041·5		65094·8	
Income	number	prop	number	prop	number	prop	number	prop	number	prop	number	prop	number	prop
< GDP per capita	206	20·2	259	25·2	211	20·6	202	20·2	494	49·1	249	24·3	396	39·6
= GDP per capita	779	76·7	734	71·5	770	75·4	748	74·8	504	50	752	73·5	593	59·3
> GDP per capita	30	2·9	33	3·2	40	3·9	50	5·0	8	0·7	21	2·0	11	1·1
Education														
Primary	8	0·7	13	1·2	25	2·4	17	1·7	17	1·6	6	0·5	3	0·3
Secondary	432	42·5	429	41·8	398	39·2	402	40·2	520	51·6	544	53·2	269	26·9
Higher/university	574	56·5	584	56·9	589	57·6	576	57·6	466	46·3	469	45·8	725	72·5
Unknown	1	0·0	0	0·0	9	0·8	5	0·5	3	0·2	3	0·2	3	0·3
Age (years)														
15–24	174	17·1	207	20·2	121	11·9	80	8·0	100	9·9	110	10·8	120	12·0
25–34	224	22·1	209	20·4	180	17·6	130	13·0	150	14·9	170	16·6	180	18·0
35–44	220	21·7	208	20·3	184	18·0	150	15·0	160	15·9	172	16·8	160	16·0
45–54	180	17·7	152	14·8	200	19·6	170	17·0	172	17·1	179	17·5	170	17·0
55–64	126	12·4	130	12·7	170	16·7	140	14·0	161	16·0	159	15·6	160	16·0
65 and above	91	9·0	120	11·7	166	16·3	330	33·0	263	26·1	232	22·7	210	21·0
Gender														
Male	522	51·9	501	48·8	499	48·8	490	49·0	480	47·3	493	48·3	488	47·6
Female	493	49·0	520	50·6	508	49·7	508	50·8	526	51·8	524	51·3	503	49·0
Non-binary	0	0·0	5	0·5	4	0·4	2	0·2	0	0·0	4	0·4	9	0·9
Others	0	0·0	0	0·0	10	1·0	0	0·0	0	0·0	1	0·1	0	0·0
Individual diagnosed of one or more health conditions														
Yes	264	26·0	354	34·5	349	34·2	326	32·6	310	30·8	343	33·6	408	40·8
No	751	74·0	672	65·5	672	65·8	674	67·4	696	69·2	679	66·4	592	59·2
Family member diagnosed of one or more health conditions														
Yes	276	27·2	342	33·3	315	30·9	290	29·0	396	39·4	429	42·0	306	30·6
No	739	72·8	684	66·7	706	69·1	710	71·0	610	60·6	593	58·0	694	69·4

GDP, gross domestic product. prop, proportion.

* GDP per capita is adjusted to purchasing power parity (PPP) obtained from World Bank 2019. Types of health conditions diagnosed are provided in Supplementary Table 4.

The gender distribution of respondents across the seven countries was relatively balanced, with marginally higher proportion of females in Brazil (52·0 %), France (51·8 %) and UK (51·3 %) (Table 1). Across all countries, approximately 30·0 % of respondents and/or their family members have been diagnosed with at least one health condition. The highest percentage was reported in the USA, where 40·8 % of respondents reported the existence of one or more health conditions. Similarly, in the UK, 42·0 % of family members of the study participants reported having one or more health conditions, which was the highest proportion among the seven countries (Table 1, Supplementary Table 4).

### Structural equation modeling regression analysis

Based on the CFA result, three observed variables were identified significantly measuring the knowledge about Na intake and eleven observed variables were significantly measuring attitude towards reducing Na intake (Supplementary Fig. 1). The details of CFA result are provided in Appendix 1·2. The SEM regression analysis, substantiated by a robust CFA, was conducted to discern the associations between socio-economic determinants, health conditions, and both knowledge and attitude across various countries.

#### Socio-economic and health determinants of knowledge and attitude related to Na intake

The influence of education on knowledge was found significant in three countries: Thailand, Indonesia and Japan with coefficient of 0·06 (95 % CI 0·04, 0·08), 0·02 (0·00, 0·04) and 0·02 (0·00, 0·04), respectively (Table 2). It means as the education level in the population increases, subsequently the knowledge about Na intake increases as well. In contrast, association of income with knowledge was found significant only in Thailand with the coefficient of 0·04 (*P*-value <0·005). This indicates that people with higher income in Thailand tends to have higher knowledge about Na intake. Individual or family health conditions were not found to have a significant association with knowledge across the majority of the countries (Table 2).

**Table 2:** Regression results of knowledge and attitude related to Na intake

Country	Knowledge about Na intake								
		∼ Income	95 % CI	∼ Education level	95 % CI	∼ Health condition	95 % CI	∼ Family health condition	95 % CI
Indonesia		0·00	–0·03, 0·01	0·02*	0·00, 0·04	0·01	0·00, 0·03	0·00	–0·01, 0·01
Brazil		0·01	0·00, 0·02	0·00	0·00, 0·01	0·00	0·00, 0·00	0·00	0·00, 0·00
Thailand		0·04***	0·02, 0·07	0·06***	0·04, 0·08	0·00	–0·01, 0·01	0·01*	0·00, 0·02
Japan		0·00	–0·02, 0·01	0·02*	0·00, 0·04	0·00	0·00, 0·01	0·01	0·00, 0·02
France		0·00	0·00, 0·01	0·00	–0·01, 0·00	0·00	0·00, 0·01	0·00	0·00, 0·00
The UK		0·00	0·00, 0·01	0·00	0·00, 0·01	0·00	0·00, 0·01	0·00	0·00, 0·00
The USA		0·01	0·00, 0·02	0·00	–0·01, 0·01	0·00	0·00, 0·00	0·00	0·00, 0·01

	Attitude towards Na intake									
	∼ Knowledge	95 % CI	∼ Income	95 % CI	∼ Education level	95 % CI	∼ Health condition	95 % CI	∼ Family health condition	95 % CI
Indonesia	0·91***	0·69, 1·13	0·00	–0·02, 0·03	0·00	–0·03, 0·01	0·01*	0·00, 0·03	−0·01	–0·02, 0·00
Brazil	1·96***	0·93, 2·99	0·01	–0·01, 0·04	0·01	–0·01, 0·03	0·01**	0·00, 0·02	0·01*	0·00, 0·02
Thailand	1·57***	1·19, 1·95	0·00	–0·04, 0·03	0·00	–0·02, 0·04	0·00	–0·01, 0·02	−0·01	–0·03, 0·00
Japan	0·94***	0·71, 1·16	0·01	0·00, 0·04	0·02*	0·00, 0·04	0·03***	0·01, 0·04	0·01*	0·00, 0·03
France	2·62***	1·45, 3·79	0·05***	0·02, 0·08	−0·02	–0·05, 0·00	0·03**	0·01, 0·05	0·02*	0·00, 0·05
The UK	2·65***	1·48, 3·82	0·08	0·05, 0·12	0·00	–0·03, 0·02	0·00	–0·01, 0·02	0·02*	0·00, 0·04
The USA	1·97***	1·21, 2·73	0·01	–0·02, 0·05	0·06***	0·02, 0·10	0·00	–0·02, 0·01	0·02*	0·00, 0·04

*** *P*-value less than 0.005; ** *P*-value less than 0.01; * *P*-value less than 0.05. Model selection was based on model statistics and confirmatory factor analysis.

While the association of income and education with attitude was minimal. The health-related factors were significantly associated with the attitude in majority of the countries. Individuals having certain health conditions such as raised blood pressure, heart diseases, strokes or other diseases exhibited tendency to have a positive attitude towards reducing or managing excessive Na intake in Japan, France and Brazil with coefficients of 0·03 (0·01, 0·04), 0·03 (0·01, 0·05) and 0·01 (0·00, 0·02) respectively. Additionally, family members having certain conditions in countries such as Brazil, Japan, the UK and the USA showed positive association with attitude, although the magnitude of the association was marginally small (Table 2).

#### Association between knowledge and attitude related to Na intake

The SEM result showed that attitude related to Na intake had a consistent positive relationship with knowledge across all nations. For instance, in the UK, a coefficient of 2·65 (95 % CI 1·48, 3·82) showed strong association between knowledge and attitude meaning individuals with higher knowledge about the Na intake showed a positive attitude towards reducing or managing excessive Na intake in their daily life. Similar trends were observed in France with a coefficient of 2·62 (1·45, 3·79) and in the USA with a coefficient of 1·97 (1·21, 2·73). However, the association between attitude and knowledge related to Na intake in Indonesia and Japan was relatively weak, with the regression coefficient 0·91 (0·69, 1·13) and 0·94 (0·71, 1·16), respectively. These findings highlight potential variances in the influence of Na-related knowledge on dietary attitudes across different cultural and national contexts.

## Discussion

The global concern surrounding excessive Na intake and its association with chronic NCD is well established^(1,24)^. Nonetheless, the exploration into the factors that shape an individual’s knowledge regarding Na intake, alongside how such knowledge influences their attitudes towards the reduction of excessive Na consumption, remains limited. Thus, our investigation contributes significantly by elucidating the impact of socio-economic and health-related factors on the knowledge and attitudes pertaining to Na intake. Furthermore, our study analyses the interplay between knowledge and attitudes across a diverse array of seven countries, thereby providing a comprehensive perspective on this critical public health issue.

Our findings highlight a consistent positive relationship between knowledge and attitude related to Na intake. This pivotal finding aligns with previous research emphasising the role of knowledge as a precursor to positive Na intake attitude^(25–27)^. However, while knowledge is a critical component, it is evident that knowledge is not the sole determinant of attitude towards Na intake. Along with the positive correlation between knowledge and attitude, other factors, especially individual health condition and family health conditions in countries such as Brazil, Japan and France, were significantly associated with Na intake attitude. This suggests that personal experiences, cultural beliefs or familial health histories might play a more influential role in shaping attitude in these countries.

Health factors, especially personal health conditions, played a pivotal role in shaping the attitude. For instance, in Brazil, Indonesia and Japan, individuals with personal health conditions were more knowledgeable about Na intake. This could be attributed to the direct experience and exposure to health information during medical consultations and treatments^(28,29)^. Furthermore, the influence of family health conditions on attitude was significant in Brazil, Japan, France, the UK and the USA. This finding was consistent with the previous studies’ findings, suggesting that family health history can influence individual health behaviours^(30,31)^. People who have family members with hypertension or other CVD may be more motivated to adopt healthier dietary behaviours, such as eating a healthy diet by all the family members.^(32,33)^ This finding holds significant public health implications as it suggests that family-based interventions may be an effective strategy to promote healthier dietary behaviours.

The influence of socio-economic factor, particularly income level on knowledge and attitude related to Na intake, was insignificant in most of the countries contrasting to the previous studies’ findings^(34–36)^. Our finding suggests that high income do not necessarily translate to the better knowledge and positive attitude and healthy dietary behaviour in individuals as suggested by a few previous studies^(34–36)^. Therefore, other cultural or systemic factors including environmental factors should be taken into consideration to thoroughly understand the association of socio-economic factors with knowledge and attitude related to Na intake.

The results from our study have significant implications for public health interventions. While initiatives like WASSH and WHO’s action plan have been established to reduce Na intake globally^(9,37)^, the lack of achievement in Na intake reduction targets among WHO member countries^(10)^ emphasise the need for effective tailored interventions. Given the diverse factors influencing knowledge and attitude across countries, interventions should be country-specific, considering socio-economic, health, environmental and cultural factors. For instance, in countries where education plays a minimal role in shaping knowledge, public health campaigns might need to leverage other platforms or community influencers to disseminate information. Fostering positive surroundings through public health campaigns and ensuring schools and workplaces offer healthy and low-Na food options to appeal individuals to make healthier choices could also prove effective. Similarly, in countries where family health condition is significantly associated with positive attitude, family-based interventions may be an effective strategy to promote health dietary and reduce excessive Na intake.

This study has several limitations. First, the study used self-reported data collection method, which may be subject to social desirability, recall and non-response biases^(38)^. For instance, the self-reported perceived amount of Na intake would be dependent on their perception and the social norms^(39)^. It means that when individuals report how much Na they consume, their estimations might be influenced by their understanding of what is considered as acceptable and healthy level of Na intake. Furthermore, the data used in this study were collected using quota sampling method which is generally accepted and convenient. However, it might not entirely capture the comprehensive representation of all segments of the population, including ethnic groups and demographic structure. Second, the study only included participants from seven countries, limiting the generalisability of the findings to other countries^(40)^. Third, the study did not assess other potential determinants of attitude, such as taste preferences, food availability and cultural factors. Finally, the SEM model assumes a linear relationship between knowledge and attitude, though this may not be true in certain circumstances^(22)^.

In conclusion, the current study provides valuable insights into the association of knowledge and attitude related to Na intake across different countries. The findings suggest that increasing knowledge and awareness about Na intake may be an effective strategy to promote healthier dietary attitudes. The complexity of the relationship between socio-economic factors and health factors with knowledge and attitude highlights the need for multifaceted and tailored approaches to reduce excessive Na intake. Future research should be conducted to explore the determinants of Na intake behaviour in detail, taking into account cultural and environmental factors, and assess the effectiveness of interventions aimed at promoting healthier Na intake attitude and behaviour. While the global challenge of excessive Na intake is increasing, understanding the intricate web of factors influencing knowledge and attitude is crucial. This study sheds light on these determinants across seven countries, providing a roadmap for more effective, tailored interventions to combat the health risks associated with high Na intake.

## Supporting information

Rauniyar et al. supplementary material Rauniyar et al. supplementary material

## Data sharing

The datasets generated during and/or analysed during the current study are not publicly available but are available from the corresponding author on reasonable request.

## Supplementary material

For supplementary material accompanying this paper visit https://doi.org/10.1017/S1368980024001551
