# Supplementary material for: Determinants of sodium intake knowledge and attitude: a cross-national analysis of socio-economic and health factors
Source: Public Health Nutr. 2024 Oct 22;27(1):e216. doi: 10.1017/S1368980024001551 (PMC11604315; doi:10.1017/S1368980024001551)
Supplement: Rauniyar et al. supplementary material [file S1368980024001551sup001.docx]

**Appendix 1.1**

**Supplementary Table 1. Questionnaire measuring knowledge about sodium intake and attitude towards sodium intake.**

A. Questions measuring latent variable knowledge about salt.

Q1: Please rate whether you think the statement is true or false:

- Q1.1 Consuming too much sodium is associated with a greater risk of heart disease and stroke.
- Q1.2 Sweet foods (e.g., cakes, candy) do not contain sodium.
- Q1.3 Cooking at home is better for controlling sodium intake.

Values: 1 – 2:

1 – True

2 – False

Q2. For each of the following statements, please rate how true or untrue each is about you:

- Q2.1 Eating too much sodium is bad for your health.

Values: 1 – 7:

1 – Not at all true of me

2

3

4 – Neither true nor untrue of me

5

6

7 – Very true of me

Q3: Thinking about age and sodium consumption, please indicate how much you agree or disagree with the following statements:

- Q3.1 Sodium is important to monitor at any age.
- Q3.2 Only those 65 and older should be concerned with monitoring sodium intake.
- Q3.3 Sodium intake should be a concern for older adults (40+) rather than those who are younger.
- Q3.4 Watching sodium intake is more important for children than adults.

Values 1 – 7:

1 – Completely disagree

2

3

4 – Neither agree nor disagree

5

6

7 – Completely agree

B. Questions measuring latent variable attitude towards salt intake.

Q4: When considering what to eat, how important is each of the following:

- Q4.1 Amount of sodium.

Values: 1 – 7:

1 – Not at all important

2

3

4 – Neither important nor unimportant

5

6

7 –Extremely important

Q5: For each of the following statements, please rate how true or untrue each is about you:

- Q5.1 A lot of foods in restaurants are too salty for me.
- Q5.2 I look for food marked “low in salt” or “low sodium”.
- Q5.3 I control how much sodium I consume.
- Q5.4 I’m not concerned about how much sodium is in the food I eat.
- Q5.5 I control how much sodium I consume.

Values: 1 – 7:

1 – Not at all true of me

2

3

4 – Neither true nor untrue of me

5

6

7 – Very true of me

Q6: For each of the following statements, please rate much you agree or disagree with each:

- Q6.1 Reducing sodium is a priority for my health.
- Q6.2 Reducing sodium is a priority for my family’s health.
- Q6.3 I avoid foods high in sodium, so I do not feel bloated or uncomfortable.
- Q6.4 I add more fresh vegetables to my diet to help lower my sodium intake.
- Q6.5 I add more fresh fruit to my diet to help lower my sodium intake.
- Q6.6 I often use reduced sodium seasonings while cooking at home.
- Q6.7 I use spices instead of salt while cooking at home.

Values 1 – 7:

1 – Completely disagree

2

3

4 – Neither agree nor disagree

5

6

7 – Completely agree

**Supplementary Table 2. Responses to the questions related to knowledge about sodium intake included in the final SEM model.**

| **Questions** | **Indonesia** | | **Brazil** | | **Thailand** | | **Japan** | | **France** | **The UK** | **The US** |
| --- | --- | --- | --- | --- | --- | --- | --- | --- | --- | --- | --- |
| **Q1.1** |  | |  | |  | |  | |  |  |  |
| 1. False | 189 (18.6) | 111 (10.8) | | 170 (16.7) | | 89 (8.9) | | 98 (9.7) | | 115 (11.3) | 166 (16.6) |
| 2. True | 826 (81.4) | 915 (89.2) | | 851 (83.3) | | 911 (91.1) | | 908 (90.3) | | 907 (88.7) | 834 (83.4) |
| **Q2.1** |  | |  | |  | |  | |  |  |  |
| 1. Not at all true of me | 12 (1.2) | | 11 (1.1) | | 6 (0.6) | | 15 (1.5) | | 21 (2.1) | 16 (1.6) | 16 (1.6) |
| 2. | 11 (1.1) | | 4 (0.4) | | 8 (0.8) | | 13 (1.3) | | 11 (1.1) | 7 (0.7) | 18 (1.8) |
| 3. | 20 (2.0) | | 11 (1.1) | | 23 (2.3) | | 26 (2.6) | | 25 (2.5) | 27 (2.6) | 24 (2.4) |
| 4. Neither true nor untrue of me | 194 (19.1) | | 61 (5.9) | | 202 (19.8) | | 155 (15.5) | | 114 (11.3) | 188 (18.4) | 114 (11.4) |
| 5 | 162 (16.0) | | 52 (5.1) | | 161 (15.8) | | 263 (26.3) | | 165 (16.4) | 221 (21.6) | 190 (19.0) |
| 6 | 227 (22.4) | | 140 (13.6) | | 192 (18.8) | | 234 (23.4) | | 285 (28.3) | 260 (25.4) | 269 (26.9) |
| 7. Very true of me | 389 (38.3) | | 747 (72.8) | | 429 (42.0) | | 294 (29.4) | | 385 (38.3) | 303 (29.6) | 369 (36.9) |
| **Q3.1** |  | |  | |  | |  | |  |  |  |
| 1. Completely disagree | 9 (0.9) | | 4 (0.4) | | 5 (0.5) | | 9 (0.9) | | 12 (1.2) | 12 (1.2) | 15 (1.5) |
| 2. | 9 (0.9) | | 6 (0.6) | | 4 (0.4) | | 5 (0.5) | | 8 (0.8) | 12 (1.2) | 7 (0.7) |
| 3. | 22 (2.2) | | 12 (1.2) | | 19 (1.9) | | 26 (2.6) | | 20 (2.0) | 24 (2.3) | 29 (2.9) |
| 4. Neither agree nor disagree | 181 (17.8) | | 61 (5.9) | | 159 (15.6) | | 219 (21.9) | | 121 (12.0) | 209 (20.5) | 163 (16.3) |
| 5 | 154 (15.2) | | 63 (6.1) | | 184 (18) | | 256 (25.6) | | 164 (16.3) | 222 (21.7) | 189 (18.9) |
| 6 | 198 (19.5) | | 110 (10.7) | | 222 (21.7) | | 193 (19.3) | | 181 (18.0) | 224 (21.9) | 212 (21.2) |
| 7. Completely agree | 442 (43.5) | | 770 (75.0) | | 428 (41.9) | | 292 (29.2) | | 500 (49.7) | 319 (31.2) | 385 (38.5) |

* Selection of the observed variables included in the table is based on the significant standardized factor loading score above 0.3 and questionnaire construct. The detail of questionnaire is provided in Table 1., UK – United Kingdom; US – United States

**Supplementary Table 3. Responses to the questions related to attitude towards sodium intake included in the final SEM model.**

| **Questions** | **Indonesia** | **Brazil** | **Thailand** | **Japan** | **France** | **The UK** | **The US** |
| --- | --- | --- | --- | --- | --- | --- | --- |
| **Q4.1** |  |  |  |  |  |  |  |
| 1. Not at all important | 7 (0.7) | 43 (4.2) | 11 (1.1) | 27 (2.6) | 45 (4.4) | 51 (5.0) | 29 (2.8) |
| 2 | 9 (0.9) | 23 (2.2) | 16 (1.6) | 33 (3.2) | 26 (2.5) | 38 (3.7) | 12 (1.2) |
| 3 | 36 (3.5) | 34 (3.3) | 46 (4.5) | 57 (5.6) | 40 (3.9) | 53 (5.2) | 49 (4.8) |
| 4. Neither important nor unimportant | 301 (29.7) | 164 (16.0) | 213 (20.8) | 360 (35.1) | 178 (17.3) | 276 (26.9) | 173 (16.9) |
| 5 | 209 (20.6) | 114 (11.1) | 175 (17.1) | 266 (25.9) | 223 (21.7) | 221 (21.5) | 211 (20.6) |
| 6 | 226 (22.3) | 186 (18.1) | 198 (19.3) | 154 (15.0) | 243 (23.7) | 207 (20.2) | 233 (22.7) |
| 7. Extremely important | 227 (22.4) | 462 (45.0) | 362 (35.3) | 103 (10.0) | 251 (24.5) | 176 (17.2) | 293 (28.6) |
| **Q5.1** |  |  |  |  |  |  |  |
| 1. Not at all true of me | 96 (9.5) | 67 (6.5) | 26 (2.5) | 38 (3.7) | 110 (10.7) | 102 (9.9) | 87 (8.5) |
| 2 | 49 (4.8) | 48 (4.7) | 19 (1.9) | 60 (5.8) | 79 (7.7) | 98 (9.6) | 52 (5.1) |
| 3 | 106 (10.4) | 64 (6.2) | 48 (4.7) | 126 (12.3) | 121 (11.8) | 129 (12.6) | 97 (9.5) |
| 4. Neither true nor untrue of me | 383 (37.7) | 238 (23.2) | 403 (39.3) | 369 (36.0) | 348 (33.9) | 305 (29.7) | 245 (23.9) |
| 5 | 143 (14.1) | 182 (17.7) | 199 (19.4) | 200 (19.5) | 173 (16.9) | 167 (16.3) | 185 (18.0) |
| 6 | 133 (13.1) | 175 (17.1) | 181 (17.6) | 134 (13.1) | 98 (9.6) | 124 (12.1) | 179 (17.4) |
| 7. Very true of me | 105 (10.3) | 252 (24.6) | 145 (14.1) | 73 (7.1) | 77 (7.5) | 97 (9.5) | 155 (15.1) |
| **Q5.2** |  |  |  |  |  |  |  |
| 1. Not at all true of me | 40 (3.9) | 51 (5.0) | 27 (2.6) | 97 (9.5) | 95 (9.3) | 105 (10.2) | 85 (8.3) |
| 2 | 34 (3.3) | 42 (4.1) | 19 (1.9) | 67 (6.5) | 53 (5.2) | 62 (6.0) | 35 (3.4) |
| 3 | 86 (8.5) | 38 (3.7) | 53 (5.2) | 129 (12.6) | 87 (8.5) | 95 (9.3) | 82 (8.0) |
| 4. Neither true nor untrue of me | 393 (38.7) | 169 (16.5) | 303 (29.5) | 306 (29.8) | 295 (28.8) | 284 (27.7) | 231 (22.5) |
| 5 | 164 (16.2) | 173 (16.9) | 191 (18.6) | 193 (18.8) | 192 (18.7) | 207 (20.2) | 192 (18.7) |
| 6 | 157 (15.5) | 185 (18.0) | 190 (18.5) | 135 (13.2) | 143 (13.9) | 142 (13.8) | 182 (17.7) |
| 7. Very true of me | 141 (13.9) | 368 (35.9) | 238 (23.2) | 73 (7.1) | 141 (13.7) | 127 (12.4) | 193 (18.8) |
| **Q5.3** |  |  |  |  |  |  |  |
| 1. Not at all true of me | 14 (1.4) | 49 (4.8) | 16 (1.6) | 157 (15.3) | 49 (4.8) | 61 (5.9) | 38 (3.7) |
| 2 | 18 (1.8) | 26 (2.5) | 10 (1.0) | 137 (13.4) | 29 (2.8) | 34 (3.3) | 24 (2.3) |
| 3 | 62 (6.1) | 66 (6.4) | 48 (4.7) | 132 (12.9) | 76 (7.4) | 81 (7.9) | 54 (5.3) |
| 4. Neither true nor untrue of me | 338 (33.3) | 186 (18.1) | 312 (30.4) | 293 (28.6) | 234 (22.8) | 287 (28.0) | 228 (22.2) |
| 5 | 182 (17.9) | 144 (14.0) | 186 (18.1) | 172 (16.8) | 222 (21.6) | 230 (22.4) | 245 (23.9) |
| 6 | 173 (17) | 192 (18.7) | 221 (21.5) | 69 (6.7) | 175 (17.1) | 170 (16.6) | 197 (19.2) |
| 7. Very true of me | 228 (22.5) | 363 (35.4) | 228 (22.2) | 40 (3.9) | 221 (21.5) | 159 (15.5) | 214 (20.9) |
| **Q6.1** |  |  |  |  |  |  |  |
| 1.Completely disagree | 17 (1.7) | 25 (2.4) | 10 (1.0) | 76 (7.4) | 83 (8.1) | 75 (7.3) | 60 (5.8) |
| 2 | 22 (2.2) | 19 (1.9) | 7 (0.7) | 59 (5.8) | 52 (5.1) | 57 (5.6) | 31 (3.0) |
| 3 | 60 (5.9) | 42 (4.1) | 23 (2.2) | 93 (9.1) | 67 (6.5) | 88 (8.6) | 53 (5.2) |
| 4.Neither agree nor disagree | 329 (32.4) | 162 (15.8) | 167 (16.3) | 344 (33.5) | 263 (25.6) | 309 (30.1) | 236 (23.0) |
| 5 | 193 (19.0) | 152 (14.8) | 215 (21.0) | 235 (22.9) | 201 (19.6) | 224 (21.8) | 218 (21.2) |
| 6 | 162 (16.0) | 181 (17.6) | 216 (21.1) | 117 (11.4) | 159 (15.5) | 120 (11.7) | 182 (17.7) |
| 7.Completely agree | 232 (22.9) | 445 (43.4) | 383 (37.3) | 76 (7.4) | 181 (17.6) | 149 (14.5) | 220 (21.4) |
| **Q6.2** |  |  |  |  |  |  |  |
| 1.Completely disagree | 17 (1.7) | 22 (2.1) | 5 (0.5) | 55 (5.4) | 76 (7.4) | 85 (8.3) | 55 (5.4) |
| 2 | 16 (1.6) | 17 (1.7) | 4 (0.4) | 44 (4.3) | 41 (4.0) | 63 (6.1) | 33 (3.2) |
| 3 | 38 (3.7) | 38 (3.7) | 23 (2.2) | 66 (6.4) | 60 (5.8) | 86 (8.4) | 62 (6.0) |
| 4.Neither agree nor disagree | 336 (33.1) | 145 (14.1) | 168 (16.4) | 328 (32.0) | 258 (25.1) | 297 (28.9) | 237 (23.1) |
| 5 | 193 (19.0) | 152 (14.8) | 195 (19.0) | 273 (26.6) | 224 (21.8) | 205 (20.0) | 216 (21.1) |
| 6 | 181 (17.8) | 196 (19.1) | 237 (23.1) | 133 (13.0) | 174 (17.0) | 144 (14.0) | 192 (18.7) |
| 7.Completely agree | 234 (23.1) | 456 (44.4) | 389 (37.9) | 101 (9.8) | 173 (16.9) | 142 (13.8) | 205 (20.0) |
| **Q6.3** |  |  |  |  |  |  |  |
| 1. Completely disagree | 23 (2.3) | 56 (5.5) | 22 (2.1) | 119 (11.6) | 98 (9.6) | 101 (9.8) | 80 (7.8) |
| 2 | 21 (2.1) | 32 (3.1) | 7 (0.7) | 75 (7.3) | 44 (4.3) | 46 (4.5) | 34 (3.3) |
| 3 | 69 (6.8) | 51 (5.0) | 31 (3.0) | 122 (11.9) | 68 (6.6) | 98 (9.6) | 73 (7.1) |
| 4. Neither agree nor disagree | 333 (32.8) | 183 (17.8) | 249 (24.3) | 395 (38.5) | 291 (28.4) | 354 (34.5) | 247 (24.1) |
| 5 | 197 (19.4) | 166 (16.2) | 250 (24.4) | 162 (15.8) | 194 (18.9) | 185 (18.0) | 184 (17.9) |
| 6 | 168 (16.6) | 189 (18.4) | 198 (19.3) | 84 (8.2) | 159 (15.5) | 126 (12.3) | 199 (19.4) |
| 7. Completely agree | 204 (20.1) | 349 (34.0) | 264 (25.7) | 43 (4.2) | 152 (14.8) | 112 (10.9) | 183 (17.8) |
| **Q6.4** |  |  |  |  |  |  |  |
| 1. Completely disagree | 9 (0.9) | 43 (4.2) | 13 (1.3) | 83 (8.1) | 78 (7.6) | 74 (7.2) | 55 (5.4) |
| 2 | 12 (1.2) | 32 (3.1) | 7 (0.7) | 60 (5.8) | 44 (4.3) | 46 (4.5) | 30 (2.9) |
| 3 | 26 (2.6) | 45 (4.4) | 18 (1.8) | 84 (8.2) | 52 (5.1) | 60 (5.8) | 62 (6.0) |
| 4. Neither agree nor disagree | 172 (16.9) | 163 (15.9) | 158 (15.4) | 315 (30.7) | 282 (27.5) | 257 (25.0) | 197 (19.2) |
| 5 | 201 (19.8) | 178 (17.3) | 242 (23.6) | 222 (21.6) | 218 (21.2) | 236 (23.0) | 237 (23.1) |
| 6 | 231 (22.8) | 183 (17.8) | 255 (24.9) | 155 (15.1) | 163 (15.9) | 166 (16.2) | 207 (20.2) |
| 7. Completely agree | 364 (35.9) | 382 (37.2) | 328 (32.0) | 81 (7.9) | 169 (16.5) | 183 (17.8) | 212 (20.7) |
| **Q6.5** |  |  |  |  |  |  |  |
| 1. Completely disagree | 6 (0.6) | 40 (3.9) | 15 (1.5) | 129 (12.6) | 113 (11.0) | 85 (8.3) | 61 (5.9) |
| 2 | 9 (0.9) | 29 (2.8) | 7 (0.7) | 99 (9.6) | 48 (4.7) | 38 (3.7) | 35 (3.4) |
| 3 | 26 (2.6) | 47 (4.6) | 23 (2.2) | 145 (14.1) | 74 (7.2) | 80 (7.8) | 71 (6.9) |
| 4. Neither agree nor disagree | 214 (21.1) | 159 (15.5) | 199 (19.4) | 349 (34.0) | 286 (27.9) | 257 (25.0) | 222 (21.6) |
| 5 | 203 (20.0) | 166 (16.2) | 232 (22.6) | 170 (16.6) | 196 (19.1) | 247 (24.1) | 220 (21.4) |
| 6 | 222 (21.9) | 187 (18.2) | 251 (24.5) | 82 (8.0) | 153 (14.9) | 160 (15.6) | 198 (19.3) |
| 7. Completely agree | 335 (33.0) | 398 (38.8) | 294 (28.7) | 26 (2.5) | 136 (13.3) | 155 (15.1) | 193 (18.8) |
| **Q6.6** |  |  |  |  |  |  |  |
| 1. Completely disagree | 26 (2.6) | 54 (5.3) | 24 (2.3) | 150 (14.6) | 99 (9.6) | 197 (19.2) | 105 (10.2) |
| 2 | 19 (1.9) | 39 (3.8) | 17 (1.7) | 108 (10.5) | 52 (5.1) | 79 (7.7) | 55 (5.4) |
| 3 | 73 (7.2) | 45 (4.4) | 44 (4.3) | 136 (13.3) | 79 (7.7) | 101 (9.8) | 64 (6.2) |
| 4. Neither agree nor disagree | 358 (35.3) | 188 (18.3) | 261 (25.4) | 349 (34.0) | 288 (28.1) | 270 (26.3) | 253 (24.7) |
| 5 | 186 (18.3) | 169 (16.5) | 250 (24.4) | 161 (15.7) | 203 (19.8) | 153 (14.9) | 194 (18.9) |
| 6 | 169 (16.7) | 201 (19.6) | 185 (18.0) | 57 (5.6) | 148 (14.4) | 116 (11.3) | 158 (15.4) |
| 7. Completely agree | 184 (18.1) | 330 (32.2) | 240 (23.4) | 39 (3.8) | 137 (13.4) | 106 (10.3) | 171 (16.7) |
| **Q6.7** |  |  |  |  |  |  |  |
| 1. Completely disagree | 36 (3.5) | 110 (10.7) | 41 (4.0) | 118 (11.5) | 68 (6.6) | 106 (10.3) | 50 (4.9) |
| 2 | 22 (2.2) | 47 (4.6) | 21 (2.0) | 86 (8.4) | 34 (3.3) | 57 (5.6) | 26 (2.5) |
| 3 | 65 (6.4) | 70 (6.8) | 51 (5.0) | 134 (13.1) | 58 (5.7) | 76 (7.4) | 56 (5.5) |
| 4. Neither agree nor disagree | 307 (30.2) | 211 (20.6) | 283 (27.6) | 337 (32.8) | 224 (21.8) | 259 (25.2) | 209 (20.4) |
| 5 | 194 (19.1) | 176 (17.2) | 251 (24.5) | 197 (19.2) | 228 (22.2) | 233 (22.7) | 228 (22.2) |
| 6 | 187 (18.4) | 183 (17.8) | 197 (19.2) | 91 (8.9) | 200 (19.5) | 158 (15.4) | 197 (19.2) |
| 7. Completely agree | 204 (20.1) | 229 (22.3) | 177 (17.3) | 37 (3.6) | 194 (18.9) | 133 (13.0) | 234 (22.8) |

* The result of only significant observed variable is provided in the table. Selection of the observed variables was based on the significant standardized factor loading score above 0.3. The detail of the questionnaire of all variables is provided in Supplementary Table 1. UK – United Kingdom; US – United States

**Supplementary Table 4. Health condition of respondents and their family members.**

|  | Indonesia | Brazil | Thailand | Japan | France | The UK | The US |
| --- | --- | --- | --- | --- | --- | --- | --- |
| Individual health condition |  |  |  |  |  |  |  |
| Hypertension | 125 (12.2) | 162 (15.8) | 135 (13.2) | 160 (15.6) | 132 (12.9) | 144 (14) | 181 (17.6) |
| Heart disease | 15 (1.5) | 11 (1.1) | 12 (1.2) | 20 (1.9) | 32 (3.1) | 19 (1.9) | 28 (2.7) |
| Stroke | 9 (0.9) | 4 (0.4) | 2 (0.2) | 1 (0.1) | 13 (1.3) | 8 (0.8) | 10 (1.0) |
| Kidney disease | 13 (1.3) | 43 (4.2) | 24 (2.3) | 22 (2.1) | 20 (1.9) | 17 (1.7) | 17 (1.7) |
| Stomach cancer | 3 (0.3) | 3 (0.3) | 12 (1.2) | 6 (0.6) | 2 (0.2) | 6 (0.6) | 2 (0.2) |
| something else | 58 (5.7) | 54 (5.3) | 33 (3.2) | 57 (5.6) | 41 (4.0) | 55 (5.4) | 45 (4.4) |
| More than one health cond. | 41 (4.0) | 77 (7.5) | 131 (12.8) | 60 (5.8) | 70 (6.8) | 94 (9.2) | 125 (12.2) |
| None of the above | 751 (73.2) | 672 (65.5) | 672 (65.5) | 674 (65.7) | 696 (67.8) | 679 (66.2) | 592 (57.7) |
| Family health condition |  |  |  |  |  |  |  |
| Hypertension | 168 (16.6) | 178 (17.3) | 160 (15.7) | 144 (14.4) | 148 (14.7) | 177 (17.3) | 178 (17.8) |
| Heart disease | 22 (2.2) | 22 (2.1) | 18 (1.8) | 15 (1.5) | 25 (2.5) | 22 (2.2) | 13 (1.3) |
| Stroke | 4 (0.4) | 12 (1.2) | 3 (0.3) | 4 (0.4) | 7 (0.7) | 10 (1.0) | 7 (0.7) |
| Kidney disease | 18 (1.8) | 18 (1.8) | 21 (2.1) | 21 (2.1) | 24 (2.4) | 27 (2.6) | 27 (2.7) |
| Stomach cancer | 3 (0.3) | 5 (0.5) | 6 (0.6) | 4 (0.4) | 1 (0.1) | 10 (1.0) | 5 (0.5) |
| something else | 49 (4.8) | 44 (4.3) | 41 (4.0) | 54 (5.4) | 60 (6.0) | 56 (5.5) | 39 (3.9) |
| More than one health cond. | 12 (1.2) | 63 (6.1) | 66 (6.5) | 48 (4.8) | 131 (13.0) | 127 (12.4) | 37 (3.7) |
| None of the above | 739 (72.8) | 684 (66.7) | 706 (69.1) | 710 (71.0) | 610 (60.6) | 593 (58.0) | 694 (69.4) |

* UK – United Kingdom; US – United States

**Supplementary Table 5. Model summary statistics structural equation model.**

| Estimator | Diagonally weighted least square (DWLS) | | | | | | |
| --- | --- | --- | --- | --- | --- | --- | --- |
| Optimization method | Nonlinear minimization using Box-constrained optimization algorithm (NLMINB) | | | | | | |
| Number of model parameters | 46 | | | | | | |
|  | Indonesia | Brazil | Thailand | Japan | France | The UK | The US |
| Number of observations | 1015 | 1026 | 1021 | 1000 | 1006 | 1022 | 1000 |
| fmin | 0.093 | 0.099 | 0.138 | 0.150 | 0.124 | 0.114 | 0.091 |
| Comparative Fit Index (CFI) | 0.993 | 0.991 | 0.985 | 0.981 | 0.986 | 0.989 | 0.994 |
| Tucker-Lewis Index (TLI) | 0.992 | 0.989 | 0.983 | 0.977 | 0.983 | 0.987 | 0.993 |
| Root mean square error of approximation (RMSEA) | 0.022 | 0.024 | 0.035 | 0.037 | 0.031 | 0.029 | 0.021 |
| Relative non-centrality index (RMR) | 0.005 | 0.005 | 0.006 | 0.008 | 0.008 | 0.007 | 0.008 |
| Goodness of fit index (GFI) | 0.991 | 0.993 | 0.994 | 0.985 | 0.988 | 0.990 | 0.993 |
| Adjusted goodness of fit index (AGFI) | 0.988 | 0.991 | 0.992 | 0.980 | 0.984 | 0.986 | 0.991 |

*fmin – minimum value of the optimizer function used in the estimation.; UK – United Kingdom; US – United States

**Appendix 1.2**

**I. Confirmatory factor analysis**

To select the significant observed variables/ items measuring the latent variables; knowledge about sodium intake and attitude towards reducing sodium intake, confirmatory factor analysis (CFA) was used. CFA belongs to the family of structural equation modeling (SEM) techniques that investigates causal relations among latent and observed variables in a priori specified theory-derived models [^19^](#_ENREF_19)^,^[^20^](#_ENREF_20). CFA provides valuable information about the fit of the data to the specific, theory-derived measurement model, where items load only on the factors they were designed to measure. It also identifies potential weaknesses of items loaded on specific factors. In CFA, load refers to the numerical values representing the relationships between observed variables (also known as items/indicators) and their respective latent variables. Factor loading scores are the estimated coefficients or weights that indicate the strength and direction of the association between each observed variable and its corresponding latent variable [^19^](#_ENREF_19)^,^[^20^](#_ENREF_20).

The significant observed variables i.e., items for the latent variable of interest related to sodium intake were selected based on the factor loading score obtained from CFA. Two latent variables of interest were considered as the outcome variables in this study: (i) knowledge about sodium intake (referred to as ‘knowledge’) and (ii) attitude towards reducing excessive sodium intake (referred to as ‘attitude’). A total of eight questions designed to measure knowledge about sodium intake, and 13 questions designed to evaluate attitude, were subjected to CFA (Supplementary Table 1). To fit the regression model in SEM, ordinal scale variables that were measuring knowledge and attitude related to sodium intake were rescaled into continuous scales between 0 and 1 reflecting the gradual increase. For the latent variable knowledge, the score closer to 0 represents lower knowledge about sodium intake meaning that the respondents have little or no information about the risk of excessive sodium intake to their health, thus being unaware. The score closer to 1 represents higher knowledge about sodium intake indicating the individuals are well informed about the risk of excess sodium intake, thus being aware. In the case of attitude, a value closer to 0 signifies a negative attitude towards reducing or managing excessive sodium intake, while a value closer to 1 signifies a positive attitude towards reducing or managing excessive sodium intake.

**Factor loadings score of knowledge about sodium intake and attitude towards reducing sodium intake based on CFA**

The significant standardized factor loading scores of observed variables to their corresponding latent variables (i.e., knowledge and attitude) are presented in Figure 2. Based on previous literature, standardized factor loading scores above 0.30 out of 1.00 were considered significant. A significant standardized factor loading score indicates strong association between the observed variable and the corresponding latent variable. Based on the standardized factor loading scores and the construct of the survey questionnaire, out of eight observed variables three were identified to be associated with knowledge. Similarly, out of 13 observed variables measuring the latent variable attitude, 11 were significantly associated. Thus, 14 observed variables were included in the final SEM for the analysis (Supplementary Figure 1 & Supplementary Table 1).

The standardized loading score for the selected observed variable (Q1.1) measuring knowledge in Brazil, France and the UK was below 0.20 (Supplementary Figure 1). This could be due to the lack of variance in the response and possible non-linear relationships between the observed variable and the latent variable. However, the variable had higher standardized factor loading score measuring sodium intake knowledge in Indonesia, Thailand, Japan, and the US hence, it was included in the final SEM analysis.


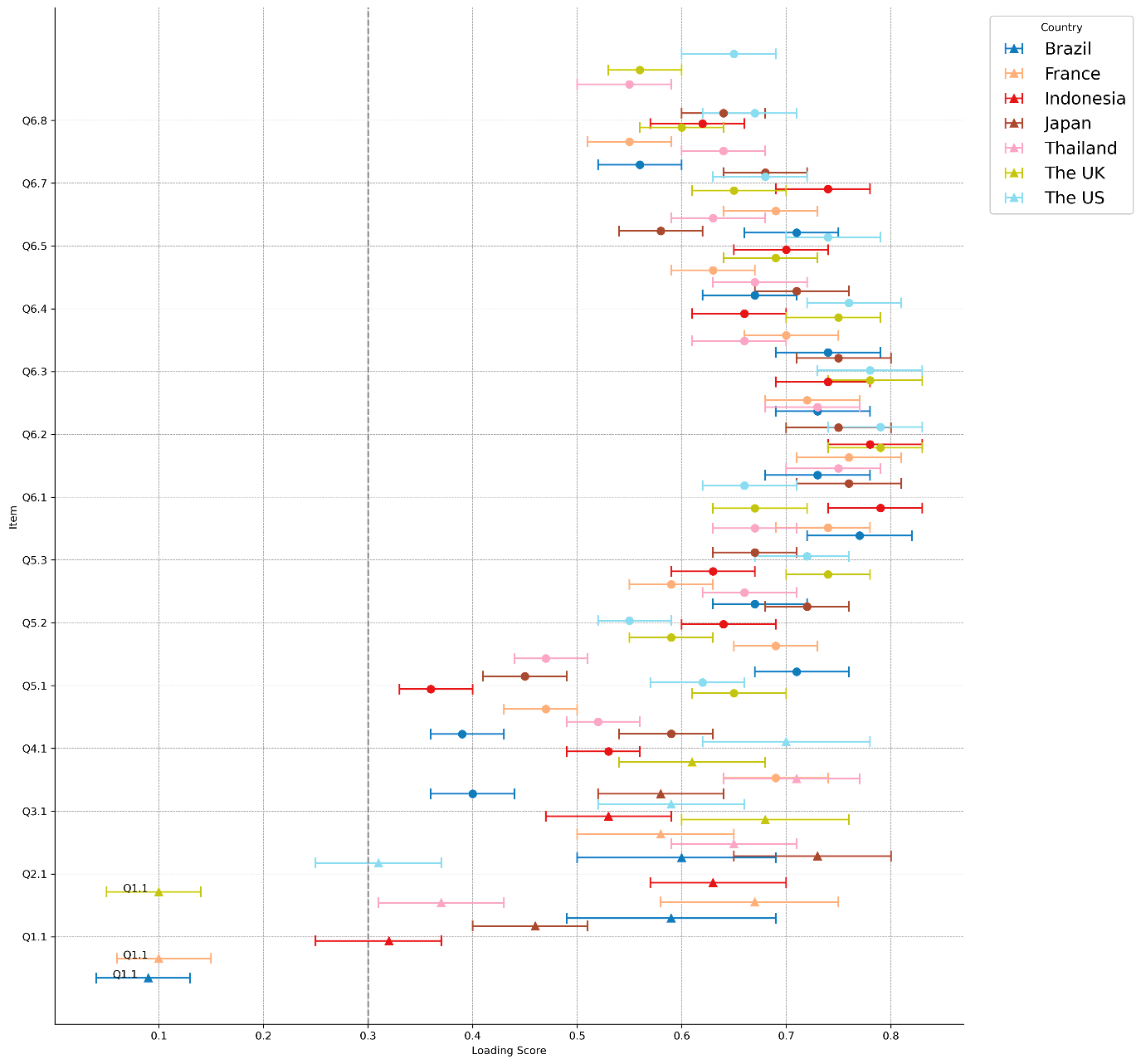


Supplementary Figure 1. Factor loading score of the observed variables to their corresponding latent variables.

* Δ indicate factor loading scores for items measuring latent variable knowledge; ○ represents factor loading score of items measuring latent variable attitude; each color represents a specific country; explanation of questions shown on y-axis is provided in Supplementary Table 1.

The CFA result indicates that the two out of three observed variables measuring knowledge had a strong relationship with the corresponding latent variable. These variables were focused on assessing the information on excessive amount of sodium intake and its effect on health (i.e., Q2.1 in the Supplementary Table 1), and the information about the relevance of monitoring sodium intake irrespective of age (Q3.1). The standardized factor loading scores for these observed variables ranged from 0.53 to 0.73 (Figure 2). For the latent variable attitude, the observed variables were focused on assessing individuals’ attitude towards the importance of sodium amount in the food (Q4.1 in Supplementary Table 1), individuals’ attitude towards the saltiness in restaurants’ food (Q5.1) attention to low-sodium labels (Q5.2), attitude towards monitoring sodium intake (Q5.3), personal and familial prioritization of reducing sodium intake for health (Q6.1 and Q6.2), and various strategies to lower sodium intake, including avoiding high-sodium foods (Q6.3), adding more vegetables and fruits to the diet (Q6.4 and Q6.5), using reduced sodium seasonings (Q6.6), and substituting salt with other species when cooking at home (Q6.7). Most of the observed variables measuring attitude towards sodium intake have high standardized factor loading scores ranging from 0.30 to 0.79 indicating a strong relationship (Figure 2). The survey response results of all the observed variables that were significantly associated with knowledge and attitude can be found in Supplementary Tables 2 & 3 respectively.

**II. Structural equation model explanation**

**Measurement model equation**

Knowledge =~ *λ*_Q1.1_ * Q1.1 + *λ*_Q2.1_ ​∗ Q2.1 + *λ*_Q3.1_ ​∗ Q3.1

Attitude =~ *λ*_Q4.1_ * Q4.1 + *λ*_Q5.1_ ​∗ Q5.1+ *λ*_Q5.2_ ​∗ Q5.2 + *λ*_Q5.3_ ​∗ Q5.3 + *λ*_Q6.1_ ​∗ Q6.1 + *λ*_Q6.2_ ​∗Q6.2 + *λ*_Q6.3_ ​∗ Q6.3 + *λ*_Q6.4_ ​∗ Q6.4 + *λ*_Q6.5_ ​∗ Q6.5 + *λ*_Q6.6_ ​∗ Q6.6 + *λ*_Q6.7_ ​∗ Q6.7

In the above equation:

*λ*_Q2.1​_, *λ*_Q3.1_​, *λ*_Q5.1_​, etc., represent the factor loadings, which quantify the relationship between the latent variable and each of its indicators.

**Structural model equation**

Knowledge ~ *b*1 ​× Income + *b*2 ​× Education + *b*3​ × Health Condition + *b*4 × Family Health Condition + *ζ*_Knowledge_​

Attitude ~ γ1 ​× Knowledge + γ2​× Education + γ3 ​× Income + γ4​× Health Condition + γ5 ​× Family Health Condition + ζ_Attitude_​

In the above equations:

Knowledge = Knowledge about sodium intake

Attitude = Attitude towards sodium intake
